# Nanoparticle-Based Techniques for Bladder Cancer Imaging: A Review

**DOI:** 10.3390/ijms24043812

**Published:** 2023-02-14

**Authors:** Federico Boschi, Manuela Malatesta

**Affiliations:** 1 Department of Computer Science, University of Verona, I-37100 Verona, Italy; 2 Department of Neurosciences, Biomedicine and Movement Sciences, University of Verona, I-37100 Verona, Italy

**Keywords:** nanotechnology, bladder cancer, urothelium, fluorescent imaging, scanning laser fluorescent microscopy, magnetic resonance imaging, optical imaging

## Abstract

Bladder cancer is very common in humans and is often characterized by recurrences, compromising the patient’s quality of life with a substantial social and economic impact. Both the diagnosis and treatment of bladder cancer are problematic due to the exceptionally impermeable barrier formed by the urothelium lining the bladder; this hinders the penetration of molecules via intravesical instillation while making it difficult to precisely label the tumor tissue for surgical resection or pharmacologic treatment. Nanotechnology has been envisaged as an opportunity to improve both the diagnostic and therapeutic approaches for bladder cancer since the nanoconstructs can cross the urothelial barrier and may be functionalized for active targeting, loaded with therapeutic agents, and visualized by different imaging techniques. In this article, we offer a selection of recent experimental applications of nanoparticle-based imaging techniques, with the aim of providing an easy and rapid technical guide for the development of nanoconstructs to specifically detect bladder cancer cells. Most of these applications are based on the well-established fluorescence imaging and magnetic resonance imaging currently used in the medical field and gave positive results on bladder cancer models in vivo, thus opening promising perspectives for the translation of preclinical results to the clinical practice.

## 1. Introduction to Urinary Bladder

The urinary bladder is a hollow organ responsible for the temporary storage of urine, which is excreted by kidneys, enters the bladder by ureters, and exits via the urethra. The bladder wall is made of three layers: the urothelium (also called the transitional epithelium), a stratified epithelium lining the bladder cavity and composed of highly specialized cells acting as a barrier to urine and pathogens, and involved in many physiological functions [1]; the detrusor muscle that is made of three differently oriented smooth muscle sheets and contracts during voiding; the external serous membrane called the adventitia.

Bladder diseases, especially those affecting the urothelium, are very common in humans; these pathologies are often characterized by recurrences, thus compromising the quality of life and having a substantial social-economic impact. Indeed, urinary tract infections are the second most common infection in women [2], while bladder cancer is one of the most frequent tumors worldwide (the second for men and the fourth for women) [3,4].

Both the diagnosis and treatment of bladder pathologies, especially cancer, are problematic due to the unique features of the urothelium, in particular to the exceptionally impermeable apical membrane of the upper umbrella cells [5]. In fact, the drugs administered by intravesical instillations hardly penetrate the urothelial barrier, thus failing to reach the diseased cells located in the lower layers of the bladder wall [6,7]. Moreover, the constant production of urine and the recurrent voiding process limit the dwell time of the therapeutic agents [8], thus making frequent administration necessary with consequent heavy local irritation. As for diagnosis, photodynamic diagnosis (PDD) by fluorescence cystoscopy is the most accredited method currently used to identify cancer cells in order to selectively resect the tumor tissue avoiding recurrence and possible radical cystectomy [9]. However, currently, PDD is based on the administration of photosensitizing prodrugs that preferentially accumulate in proliferating urothelial cells but do not enter specifically urothelial cancer cells, thus rising important limitations in its reliability [10,11].

## 2. Nanotechnology and Urinary Bladder Imaging

In this context, nanotechnology has been envisaged as a great opportunity to improve both diagnostic and therapeutic approaches for bladder diseases. In fact, nanoconstructs are able to cross the urothelial barrier due to their small size approaching the molecular scale, may be functionalized for active targeting, can be visualized by different imaging techniques based on their physico-chemical features, and may be loaded with therapeutic agents, thus ensuring sustained release at the target site [12,13,14,15,16,17,18]. Nanoparticles (NPs) have also been explored as diagnostic tools to quantify analytes in urinary samples [19] and have been used for regenerative medicine and tissue engineering to enhance bladder repair/reconstruction and functional recovery [20].

To develop novel nanoconstructs suitable for applications on the urinary bladder, it is crucial to assess their uptake and distribution in healthy and diseased bladder cells and tissues. To this aim, imaging techniques proved to be unique in tracking the NPs in experimental bladder models in vitro and in vivo (e.g., [21,22,23,24]). In particular, in vivo administration of NPs for bladder cancer targeting may be performed by instillation into the organ via urethra or by intravenous (i.v.) injection and, especially in the latter case, monitoring their biodistribution is essential to assess their efficacy and safety. Therefore NPs, designed not only to target bladder cancer cells but also to be effectively visualized by different imaging techniques, represent a significant advantage for the development of novel diagnostic and therapeutic strategies.

The most used imaging techniques in preclinical research aimed at setting up diagnostic and therapeutic nanoconstructs or intraoperative nanotools are based on fluorescence [25,26]. NPs can be made visible by modulating their physico-chemical features as it occurs for quantum dots or nanodiamonds [27,28,29] or by loading with appropriate fluorophores (some examples in [30,31,32,33]). However, special attention must be paid to the selection of fluorescent NPs with appropriate spectral characteristics, especially when they are administered in vivo. In fact, in biological tissues, only a small absorption window in the near-infrared wavelength range, between 650 nm and 900 nm, is useful for in vivo image acquisition due to the hemoglobin absorption on one side and the water and lipid absorption on the other side of the spectral range. This range is further limited between 650 and 750–800 nm (called near-infrared I, NIR I) due to the sensitivity of the detectors used in confocal laser scanning microscopy (CLSM) or in whole-body/whole-organ optical imaging (OI) (i.e., the photomultipliers or the charge-coupled device (CCD), respectively) [34]. Detector improvements and extended sensitivity presently allow exploiting another NIR region (named NIR II) of an almost optically transparent tissue window in the 1000–1700 nm wavelength range. The interest in the NIR II window has re-focused attention on tissue autofluorescence, resulting from the emission by tissue or organs occurring when excited with external sources. Most endogenous fluorophores accumulate in animal organs with feeding and emit in the blue/green part of the wavelength region of the electromagnetic spectrum. To avoid overlapping with autofluorescence, it is necessary to shift to red or NIR the emission of the NPs in order to make them detectable. However, a recent study reported that autofluorescence affects in vivo imaging even in the NIR II window [35]. To overcome this problem, upconverting NPs can be used, which are excited by multiple photon interactions to produce a single photon with energy greater than each incoming photon; upconverting NPs are especially convenient since very few organic molecules with upconverting optical properties exist in the living tissues [36,37,38]. 

Despite the limitations due to the optical tissue absorption, which reduces the number of photons passing through the tissues, and the scattering, which deflects the trajectory of the photons, fluorescence imaging (FLI) is a very fast, cheap, and easy technique that is largely used in many laboratories. For bladder diseases, FLI is especially suitable for cystoscopic analyses due to the small thickness of the urothelium that facilitates the detection of the fluorescence signals; on the contrary, FLI has a limited application to whole-body detection due to the absorption by the abdomen wall tissues covering the bladder.

FLI is especially used for preclinical applications, while another technique, magnetic resonance imaging (MRI), is largely used also in the clinical field. 

MRI takes advantage of the high resolution of the images and three-dimensional (3D) tomographic reconstruction, which is generally unsuitable for FLI. In recent years, the availability of increasing magnetic field intensity significantly improved image quality. Moreover, the 3D measure of tumor volume makes it possible to stage the cancer or monitor the response in terms of mass reduction following therapeutic treatments [39,40,41,42]. Many NPs have been developed to be used in MRI as contrast agents or diagnostic biosensors, for hyperthermia therapy, or for targeted drug or gene delivery [43,44,45,46]. These nanoconstructs are mostly based on iron oxide or gold and are characterized by magnetic properties that make them easily detectable in a biological environment that virtually lacks magnetic background; moreover, they can be made biocompatible by appropriate binding or coating, and their surface can be functionalized for specific targeting. Thanks to these properties, NP-based MRI has also been considered a promising tool for bladder cancer diagnosis and treatment [47,48].

Due to the increasing interest in nanotechnology for bladder cancer pathology, in this work, we offer a selection of recent experimental applications of NP-based imaging techniques to this research and clinical field. Several NPs have been constructed to target bladder cancer cells, but in the present review, we selected only those designed for detection by imaging techniques currently used in the medical field (mainly FLI and MRI), with the aim of providing an easy and quick technical guide, especially for scientists involved in the development of nanoconstructs to specifically detect bladder tumor for diagnostic and therapeutic purposes.

## 3. Quick Overview of the Literature

Four tables containing a selection of interesting references on the synthesis or application of NPs to bladder cancer are presented in this section: Table 1 contains articles on NPs suitable for FLI, Table 2 on NPs for MRI, Table 3 on NPs suitable for bimodal imaging (both FLI and MRI), and Table 4 on NPs suitable for other imaging techniques. The list is focused on studies explicitly devoted to biomedical imaging. Some other contributions were added due to their applications in photodynamic therapy (PDT) or PDD if the imaging aspect was relevant.

The information reported in the columns of the table is listed below.

1. Nanoparticles: NPs or NP-based compounds tested by the authors. Brief description of the particles and abbreviations used throughout the papers by the authors. When reported, the size of the NPs is given. The instrument used to measure NP size, i.e., transmission electron microscopy (TEM), dynamic light scattering (DLS), or atomic force microscopy (AFM), is also reported. The size of NPs is not reported if they are inserted into a more complex system; in this case, the overall size of the system is reported;
2. Imaging techniques: The techniques used for imaging purposes are reported, generally FLI (CLSM or OI) or MRI. Bioluminescent imaging was also used to monitor tumor growth. Few details regarding the detector/tomograph are reported, and, for FLI, the excitation and emission wavelengths used;
3. Experimental design: Cancer model, in vitro, in vivo, or ex vivo experimentation, route of NPs administration, animal species, NP concentration, and incubation/visualization time;
4. Main results: Among the results reported by the authors, particular attention was focused on the imaging results and other observations considered relevant for the readers.

## 4. Technical Clues for Nanoparticle-Based Imaging of Bladder Cancer Cells

The studies reported in Table 1, Table 2, Table 3 and Table 4 provide useful information to overcome various technical issues to be faced in planning reliable NP-based imaging techniques suitable for the specific detection of bladder cancer cells. Below, a summary of the successful approaches is reported.

### 4.1. Nanoparticles

**Figure 1:**
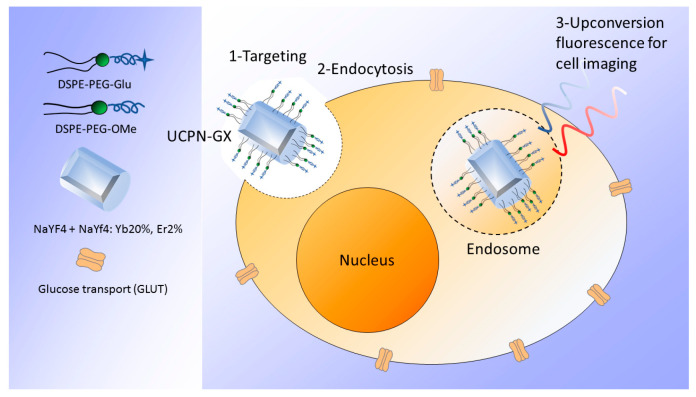
Glycosylated phospholipid-coated upconverting NPs internalized by bladder cancer cells and source of upconverting luminescence under NIR (980 nm) irradiation (adapted from [59]).

A large variety of NPs proved to be suitable for bladder cancer detection by applying FLI, MRI, bimodal FLI-MRI, or other imaging techniques. In particular, for FLI, quantum dots [49,50,52,65], mesoporous silica NPs [22,62], and titanium dioxide NPs [54] were frequently used. To increase the luminescent signal-to-noise ratio, upconverting NPs [53,59] (Figure 1) proved to ameliorate the detection of bladder cancer cells.

**Figure 2:**
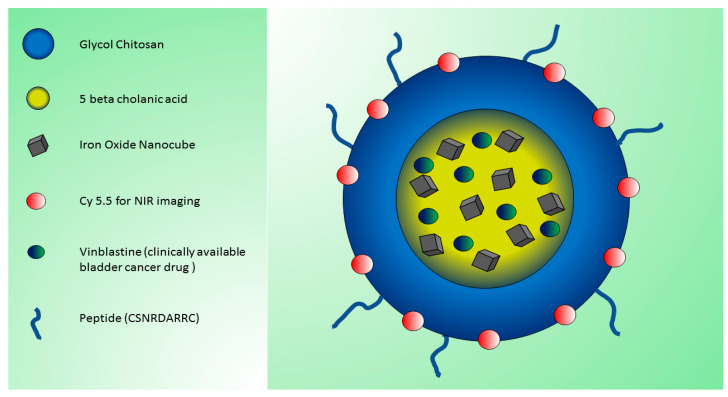
Chemical structure of glycol chitosan NPs conjugated to hydrophobic 5β-cholanic acid, a bladder cancer-targeting peptide (CSNRDARRC), iron oxide nanocubes, and the anticancer drug vinblastine. Cy5.5 was chemically conjugated to the glycol chitosan for NIR fluorescence imaging (adapted from [66]).

For MRI, contrast agent NPs were composed of iron oxide [61,63,66] (Figure 2), manganese dioxide [67], or ferrous selenide [64].

Albumin-based nanoplatforms [57,67], single-walled carbon nanohorns [50], gold nanorods [53], titanium dioxide [54], and silica NPs [56] loaded with fluorescent or magnetic agents or even with other NPs were also employed for imaging purposes.

Aggregation-induced emission (AIE) is the process in which weakly luminescent molecules become very bright by aggregating. AIE luminogens are, therefore, interesting molecules suitable to synthesize organic NPs for biomedical imaging thanks to their brightness. Accordingly, AIE NPs were applied in some studies for bladder cancer detection [51,60].

### 4.2. Imaging Techniques and Detectors

**Figure 3:**
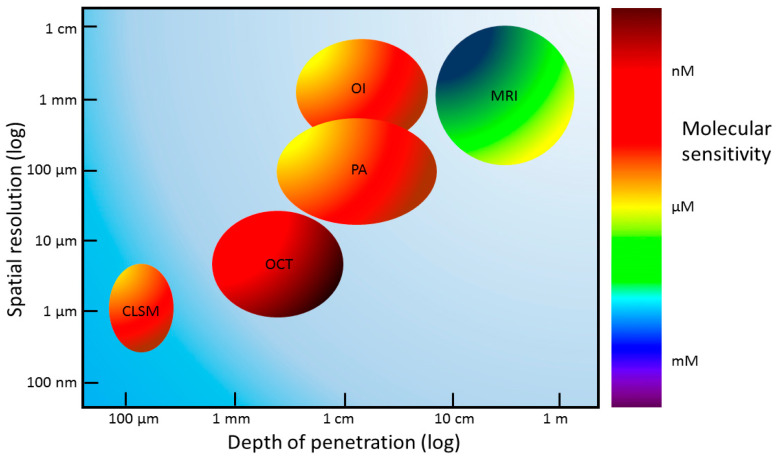
Schematic representation of the spatial resolution, depth of penetration, and molecular sensitivity of different imaging techniques. The colors indicating molecular sensitivity are not related to the x- and y-axis values. CLSM, confocal laser scanning microscopy; OCT, optical coherence tomography; OI, optical imaging; PA, photoacoustic imaging; MRI, magnetic resonance imaging.

The most used techniques to investigate bladder cancer by NPs detection are differently suited to morphological tissue imaging (Figure 3). 

MRI, OI, and photoacoustic (PA) imaging are largely diffused techniques that allows whole-body or whole-organ imaging, but they have low-resolution capabilities (fraction of a millimeter). Instead, CLSM can provide high-resolution images (below 1 micrometer), thus revealing sub-cellular structures, but it penetrates less deeply into the sample, reaching 100 μm in depth. Optical coherence tomography (OCT) can achieve a spatial resolution of 10 μm and a few micrometers in depth [71,72].

MRI has a molecular sensitivity in the range of micromolar–millimolar, while PA, OI, and CLSM can obtain nanomolar sensitivity. Recently it has been demonstrated that OCT can achieve picomolar sensitivity for in vivo imaging [73,74].

In the articles selected in this review, conventional fluorescence microscopy and CLSM were often employed to visualize in vitro and to confirm ex vivo the uptake of fluorescent NPs in bladder cancer cells [22,50,52,53,54,57,58,59,60,63]. For whole-body detection in both orthotopic and subcutaneous cancer models, the most useful approach was whole-body or whole-organ OI, generally conducted with IVIS^®^ instruments or analogs, which are equipped with very sensitive CCD cameras, often cooled to reduce the thermal noise inside the electronic detector [22,55,56,57,58,60,66,67]. 

**Figure 4:**
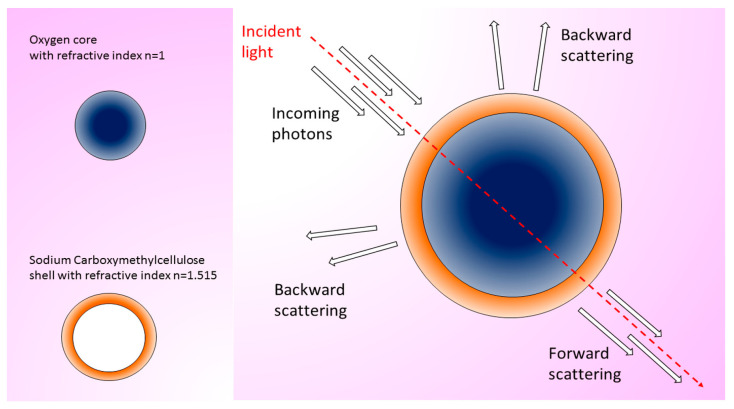
Oxygen nanobubbles (ONBs) are composed of an oxygen core and a sodium carboxymethylcellulose shell with large nonuniformities in the refractive index range, useful to obtain a high scattering signal from the probes and images with high signal-to-noise ratio. The HSI allows the acquisition of a high-resolution spectrum in each pixel in an image. From the collected spectral signatures, the localization of the ONBs can be obtained (adapted from [68]).

Hyperspectral imaging (HSI) collects a high-resolution spectrum at each pixel, allowing identification of the location of the NPs with great accuracy thanks to their spectral signatures. Dark-field microscopy, excluding the unscattered incident beam, generates a clear background in the images, which enhances the contrast. Interestingly, HSI, in combination with dark-field microscopy, allowed the successful tracking of single oxygen nanobubbles in vitro and ex vivo in bladder cancer tissue [68] (Figure 4).

Cancer growth was detected in the whole body by bioluminescent imaging techniques using luciferase-expressing (Luc+) cells [58].

In the case of MRI, tomographs with magnetic fields from 1.5 T to 9.4 T were used [22,61,62,63,64,65,66,67]. MRI was especially useful in detecting in vivo NPs injected i.v. in mice, allowing us to monitor their biodistribution and tumor-targeting efficacy. 

Interestingly, bimodal NPs suitable for both FLI and MRI were developed in order to combine the information given by the two imaging techniques, making these nanoconstructs suitable for in vitro, in vivo, and ex vivo analyses [22,65,66,67].

### 4.3. Cancer Models and Targeting

Different cancer cell lines were used as in vitro cancer models. In particular, transitional cancer cells MB49 were largely employed [22,55,57,62,67,68,69], but also human transitional carcinoma cells UMUC3 [51,54,58,59] and human bladder urothelial carcinoma EJ cells [52,56,63,64] were treated with NPs for imaging purposes. Moreover, a cancer model was obtained by injecting mice i.v. with N-butyl-N-(4-hydroxybutyl)-nitrosamine (BBN) [60].

In vivo, both subcutaneous [22,55,56,57,58,60,61,64,66,69] and orthotopic [51,62,67,70] murine models of bladder cancer were adopted. It should be underlined that although the subcutaneous model is relatively simple to obtain in animals with standardized procedures, the model closer to the pathological target in humans is the orthotopic one, where cancer cells are implanted in the organ/tissue matching the tumor histotype. In most cases (especially in subcutaneous cancer models) NPs were administered i.v. [51,55,56,57,60,61,64,66,67,69,70] to verify their capability to systemically reach tumor cells. Alternatively, NPs were instilled in the bladder in order to assess their capability to cross the urothelial barrier [22,62,67]. Taking into account that the final scope of this experimentation is setting up an NP-based imaging system to target cancer cells in the urinary bladder and that this hollow organ can be easily reached in a non-invasive way through the urethra, the administration of NPs by instillation into the bladder seems more promising for a clinical application in the near future, both for diagnostic and therapeutic purposes. The use of appropriate NPs would overcome the problem of crossing the urothelial barrier, and the use of a cystoscope associated with a suitable light source would allow the immediate visualization of the labeled tumor tissue. Accordingly, some ex vivo studies were performed by installing the NPs into intact human bladders obtained from patients who underwent radical cystectomy [49,56,63]. In other studies, ex vivo analyses were performed on major organs or tumors excised from animals treated in vivo with NPs to confirm their biodistribution [22,55,57,58,60,66,67,68,69,70]. 

To increase the specific internalization of NPs into bladder cancer cells, different strategies were adopted, e.g., the use of peptides [62,63,66], antibodies against molecules overexpressed in bladder cancer cells [49,52,53,61], or plasmids [58].

**Figure 5:**
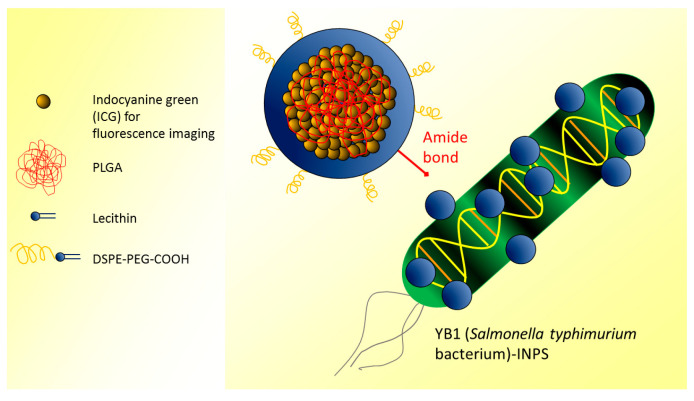
Nanophotosensitizers (indocyanine green-loaded PLGA NPs) were covalently attached to the surface of YB1 (*Salmonella typhimurium* bacterium) (adapted from [55]).

An original approach involved the genetically modified *Salmonella typhimurium* strain YB1, which is known to penetrate hypoxic tumor cores avoiding damage to normal tissues. It is, in fact, essential not only to kill tumor cells but also to preserve the surrounding healthy tissue [75]. Nanophotosensitizers indocyanine green-loaded NPs were covalently attached to the surface of YB1, and the YB1-INPs treatment demonstrated specific hypoxia-targeting to solid tumors with high fluorescence emission [55] (Figure 5).

## 5. Conclusions

The treatment of bladder cancer has become a central target for the current clinical research due to the high incidence of this tumor and its high social and economic costs in terms of low quality of life, disability, and mortality. To obtain tumor eradication, bladder cancer cells must be detected by specific markers (to allow their precise location for surgical removal) or targeted by therapeutics (for pharmacological treatment). However, the bladder urothelium represents an almost impenetrable barrier that hinders the administered molecules from reaching cancer cells. To overcome this crucial limitation, nanotechnology has been envisaged as a possible solution. In fact, thanks to their nanometric size, nanoconstructs can cross the urothelial barrier; moreover, their surface can be functionalized to specifically bind cancer cells and act as targeted therapeutic tools by delivering antitumor agents or as highly specific diagnostic tools provided that they are detectable by imaging techniques.

During the last decade, researchers have intensely been working to develop NP-based techniques to specifically image bladder cancer cells. The selected articles reported in the present review are good examples of the current ingenious experimental proposals of NP-based imaging techniques. However, the various developed nanoconstructs are quite heterogeneous, and most of them have been tested in preclinical cancer models weakly correlated with the diagnostic or therapeutic process in human patients. Therefore, at present, none of the proposed strategies seem to be ready for translation to clinical practice.

On the positive, most of the attempts are based on the well-established FLI and MRI that are currently used in the medical field. FLI is very useful in preclinical studies, but due to tissue absorption, its use in clinics would be restricted to the direct application on the bladder wall in PDD and PDT. However, this should not be seen as a limitation because the instillation of drugs directly into the bladder is a widely applied administration procedure. The advent of NPs crossing the urothelial barrier and targeting cancer cells would represent an upgrade rapidly applicable to the current clinical procedures. MRI is a powerful tool to investigate the biodistribution of NPs in vivo, especially when they are administered systemically by i.v. injection; MRI also allows monitoring of the tumor 3D features following therapeutic treatment. However, the systemic administration of NPs suitable for MRI is still unauthorized in clinical practice. Therefore it is likely that the translation of this approach from preclinical research to patient treatment will take a long time.

In sum, this review demonstrates that several original nanoconstructs proved to be suited to the targeted imaging of bladder cancer cells, but their clinical applicability still seems far away. However, these promising results represent a solid experimental foundation to proficiently pursue the efforts to set up novel NP-based diagnostic and therapeutic tools and increase the success rate in combating bladder cancer.

## Figures and Tables

**Table 1:** Studies focusing on imaging bladder cancer tissue or cells using NPs or NP-based compounds designed for FLI. Studies are listed chronologically from the earliest to the most recent. AIE, aggregation-induced emission; CLSM, confocal laser scanning microscopy; DLS, dynamic light scattering; exc, excitation; ems, emission; OI, optical imaging; PEG, polyethylene glycol; PDT, photodynamic therapy; PDD, photodynamic diagnosis; QD, quantum dots; TCC, transitional cancer cells. TEM, transmission electron microscopy.

Nanoparticles	Imaging Technique	Experimental Design	Main Results	Ref.
QD 625 conjugated with antibody anti-CD47	Clinical confocal endomicroscopy system based on a 2.6 mm fiber optic probe with a microscopic field of view (240 mm) and acquired video sequences at 12 frames/s.	Humans: QD 625 were instilled into fresh, intact bladders obtained from human subjects after radical cystectomy for muscle-invasive or high-risk non-muscle-invasive bladder cancer.	Successful endoscopic imaging of human bladder cancer cells by targeting the protein CD47, highly expressed in a variety of cancer cells but undetectable in normal urothelium.	[49]
Single-walled carbon nanohorns QD-cisplatin (SWNH-QD+cis) TEM: 337 ± 11 nm	Fluorescence microscopy.	In vitro: rat AY-27 cancer cells. Incubation time: 1 h. Visualization time: 24, 48, and 72 h.	SWNH-QD+cis were well-trackable for 3 days. SWNH-QD+cis efficiently releases cisplatin in vitro.	[50]
BPN-BBTD AIEgen encapsulated into amphiphilic polymer NPs (BPN-BBTD NPs) TEM: 37.1 ± 2.3 nm	FLI: whole-body imaging in NIR II window and NIR I window. In particular: NIR II window using 785 nm laser beam InGaAs camera, a 1000 nm long-pass filter. NIR I window: exc 700, ems < 900 nm by IVIS^®^ Spectrum.	In vivo: nude mice with subcutaneously xenografted bladder tumors or orthotopic bladder tumors (human UMUC3 cancer cells) were injected i.v. with BPN-BBTD NPs. Incubation/visualization time: 1 h.	BPN-BBTD NPs were capable of monitoring subcutaneous and orthotopic tumors for a long time (32 days).	[51]
QDs605 conjugated with an antibody against the prostate stem cell antigen (QD-PSCA).	Fluorescence microscopy.	In vitro: human EJ cancer cells. Concentration: 10 nM. Incubation time: 30 min. Visualization time: 6, 24, and 48 h.	QD-PSCA was able to specifically recognize the PSCA protein expressed in bladder cancer cells; fluorescence was stable and long-lasting.	[52]
Upconverting NPs coupled with gold nanorods conjugated with an antibody to epidermal growth factor (EGF) receptor (UCNP-AuNR nanocluster) TEM: 48.2 ± 5.17 nm	CLSM (exc 980 nm)	In vitro: human T24T cancer cells. Concentration: 8 × 10^10^/mL. Incubation/visualization time: 1–2 h.	High contrast imaging and high sensitivity detection of bladder cancer cells. Nanobubbles forming in the vicinity of the AuNRs after irradiation by a femtosecond pulsed laser were able to disrupt the cell membrane (useful for PDT).	[53]
Alexa-PEG-modified titanium dioxide NPs (TiO_2_-PEG NPs) DLS: 123.8 nm	Super-resolution fluorescence microscopy.	In vitro: human UMUC3 TCC. Incubation/visualization time: 1, 2, and 4 h.	The high uptake by bladder cancer cells led to the intracellular accumulation of TiO_2_-PEG NPs, thus increasing their fluorescence.	[54]
Indocyanine green-loaded PLGA NPs covalently attached to YB1 (*Salmonella typhimurium* bacterium) (YB1-INPs)	FLI: OI by CRi Maestro (exc 704 nm, ems 735 nm)	In vivo: mice were subcutaneously injected with mouse MB49 cancer cells and injected i.v. with YB1-INPs. Concentration: 107 µg/mL. Incubation/visualization time: 12 h. Ex vivo: analysis of tumor sections. Incubation/visualization time: 72 h.	YB1-INPs acted as nanophotosensitizers leading to specific hypoxia and perfect photothermal conversion, targeting solid tumors, and showing efficient fluorescence imaging properties.	[55]
SiO_2_ NPs and liposomes labeled by cyanine (named tumor-selective cascade activatable self-detained system-TCASS)	FLI: OI by Maestro II and IVIS^®^ Spectrum CT	In vivo: nude mice with xenografted EJ urothelial cancer cells were injected i.v. with NPs. Concentration: 14 mg/kg. Incubation/visualization time: from 1–120 h (Maestro II); from 2–48 h (IVIS). Humans: NPs instilled in intact excised human bladders. Concentration: 50 µM. Incubation/visualization time: 1 h.	The in vivo self-assembled molecules, combined with the NIR probe, showed high specificity and sensitivity for detecting bladder cancer cells.	[56]
BSA-multifunctional BITT@DSP NPs with an albumin-based NP decorated with the cisplatin (IV) prodrug and loaded to produce strong NIR FLI (BSA-BITT@DSP NPs) TEM and DLS: 70.2 ± 22.0 nm	FLI: CLSM In vivo OI by ChemiDoc MP imaging system (exc 647, ems 695 nm)	In vitro: mouse MB49 cancer cells. In vivo: mice bearing subcutaneous MB49 tumors were injected i.v. with BITT@BSA−DSP NPs. Incubation/visualization time: from 2–10 min. Ex vivo: analysis of excised organs. Incubation/visualization time: 6 h.	BITT@BSA−DSP NPs were efficiently taken up by bladder cancer cells both in vitro and in vivo.	[57]
Cationic polymer mucoadhesive EAGC-DOPE hybrid lipid NPs (EGCDNPs) complexed with Cy5-GFP-pDNA or FLuc-pDNA TEM: 30 ± 13 nm (EGCDNPs); from 67 ± 15 to 98 ± 28 nm (complexes)	FLI: CLSM Bioluminescent imaging: OI by IVIS^®^ Spectrum.	In vitro: human UMUC3 and TCC-SUP bladder cancer cells; human PC-3prostate cancer cells; human U87-MG glioblastoma cells; human HEK-293 T embryonic kidney cells were treated with EGCDNPs—Cy5-GFP-pDNA complexes. In vivo: EGCDNPs—Cy5-GFP-pDNA complexes were instilled in the bladder of healthy mice. Concentration: 2.4 or 6.4 µg/mouse. Incubation/visualization time: 24 and 48 h. Ex vivo: bladder tissue was analyzed immunohistochemically for Luc detection.	Tuning the molecular weight of the mucoadhesive cationic polymer in NPs increased gene transfer by improving adherence and penetration through the bladder barrier.	[58]
Glycosylated PEGylated phospholipid upconverting NPs oleic acid-capped NaYF4: Yb 20%, Er 2%@NaYF4 core–shell structured with phospholipid mixture (X = 0, 25, 50, 75, or 100). (UCNP-GX) DLS: from 42.3 to 60 nm, depending on the phospholipid mixture	Multiphoton fluorescence imaging (exc 908 nm, ems 545 nm)	In vitro: UMUC3 cells. Concentration: 20 or 80 µg/mL. Incubation/visualization time: 2 h.	UCNP-G100 improved the contrast between bladder cancer and normal cells. For PDD, these NPs may be used together with a cystoscope equipped with a NIR light source.	[59]
AIE molecules obtained by incorporation of the tetraphenylethylene unit to the triazaborolopyridiniu-m encapsulated within phospholipid- connected PEG (TT-1@DSPE-PEG) DLS: 80.7–83.7 nm	FLI: CLSM (exc 488 nm, ems 550–590 nm and OI by IVIS^®^ Spectrum (exc 500 nm, ems 560 nm)	In vitro: human H1299 lung cancer cells. Concentration: 5 or 10 mM. Incubation/visualization time: 2 h. In vivo: BBN-driven bladder cancer model mice were injected i.v. with TT-1@DSPE-PEG. Concentration: 40 mM. Incubation/visualization time: from 5 min to 4 h. Ex vivo: analysis of excised major organs.	NPs showed bleft red fluorescence within the cells after a short incubation time. The increased fluorescence signal observed ex vivo in the tumor and intestine of treated mice indicated NP accumulation.	[60]

**Table 2:** Studies focusing on imaging bladder cancer tissue or cells using NPs or NP-based compounds designed for MRI. Studies are listed chronologically from the earliest to the most recent ones. AFM, atomic force microscopy; DLS, dynamic light scattering; PEG, polyethylene glycol; SPIO, superparamagnetic iron oxide; TCC, transitional cancer cells. TEM, transmission electron microscopy.

Nanoparticles	Imaging Technique	Experimental Design	Main Results	Ref.
Nanoplatform SPIO with phosphonate group (PO)-PEG and antibody against ET_A_ receptor labeled with Alexafluor 488 (γFe_2_O_3_@PO-PEGx-Ab-AF488) TEM: 9.6 nm	MRI (7 T)	In vivo*:* mice injected i.v. with NPs. Concentration: 200 µM Fe/kg	Efficiency of the antibody to target specifically ET_A_ receptor overexpressed on different bladder cancer cells. The high r2/r1 ratios confirmed the great potential of these NPs as T2-shortening contrast agents for contrast-enhanced MRI applications. Labeling with Alexafluor 488 made these NPs potentially useful for bimodal imaging (MRI and FLI).	[61]
Cyc6-functionalizedMesoporous Silica NPs (Cyc6-FITC-Gd2O3-MSN) DLS: 187.3 nm	MRI (4.7 T)	In vivo: MSN instillation into the bladder of mice bearing Luc+ murine MB49 TCC and human T2442 TCC orthotopic tumor. Incubation/visualization time: 6–8 days.	Enhanced T1- and T2-weighted MRI signals, improving the detection of the tumor boundaries. Cyc6 peptide improved binding efficiency and specificity to bladder cancer cells.	[62]
Nanoscale oxygen generator (PLZ4@SeD) encapsulating SPIO NPs and organoselenium with PLZ4 peptide for bladder cancer targeting (PLZ4@SeD) TEM: ~150 nm	MRI (1.5 T)	In vitro: human EJ cancer cells. Concentration: until 4 µM. Incubation/visualization time: from 1 to 8 h. Humans: PLZ4@SeD was instilled into bladders from patients after the radical cystectomy. Concentration: 1 mM. Incubation/visualization time: from 1–8 h.	PLZ4@SeD precisely targeted the tumor inside the bladder and enhanced the T2 MRI contrast	[63]
Black phosphorus nanosheets covalently bond with SPIO selenide to construct heteronanostructure NPs modified with methoxy PEG (mPEG-NH_2_) (BPs-FeSe_2_-PEG) AFM: ~10 nm	MRI (9.4 T)	In vitro: human EJ cancer cells. Concentration: until 0.02 mM. In vivo: BPs-FeSe_2_-PEG were injected i.v. in nude mice with a subcutaneous cancer model. Concentration: 10 mg/kg. Incubation/visualization time: from 2 to 24 h.	BPs-FeSe2-PEG acted as a T2 MRI contrast agent. NPs enhanced photothermal conversion efficiency and photostability to realize MRI-guided PTT.	[64]

**Table 3:** Studies focusing on imaging bladder cancer tissue or cells using NPs or NP-based compounds for bimodal imaging (both FLI and MRI). Studies are listed chronologically from the earliest to the most recent ones. DLS, dynamic light scattering; exc, excitation; ems, emission; OI, optical imaging; PEG, polyethylene glycol; PDT, photodynamic therapy; QD, quantum dots; SPIO, superparamagnetic iron oxide; TCC, transitional cancer cells. TEM, transmission electron microscopy.

Nanoparticles	Imaging Technique	Experimental Design	Main Results	Ref.
QD-capped magnetite Nanorings (QD-FVIO) TEM: 210 and 100 nm DLS: 310 and 155 nm	Bimodal imaging: FLI (two-photon microscopy exc 756 nm, ems long pass 560 nm) and MRI (1.5 T)	In vitro: MGH cancer cells. Concentration: 0.05 mg/mL. Incubation and visualization: 24 h.	QD-FVIO’s r2* relaxivity and r2*/r1 ratio were 4 times and 2 orders of magnitude, respectively, greater than those of commercial SPIOs (ferucarbotran). The uptake and intracellular fate of QD-FVIOs were monitored	[65]
Bimodal Mesoporous Silica NPs (PEG-TRITC-Gd_2_O_3_-MSN) DLS: 80–180 nm	Bimodal imaging: FLI (OI by IVIS^®^ 200) and MRI (4.7 T). Fluorescence microscopy	In vitro: Luc+ murine MB49 TCC and human T24 TCC. In vivo: (1) Subcutaneous injection of TCC labeled with MSN or instillation into the bladder of mice. Concentration: 1 × 10^5^ cells. (2) Installation of free MSN into the bladder after tumor development. Concentration: 5 × 10^5^ cells Ex vivo: Microscopy analysis of excised bladders.	High cell uptake of MSN. MRI revealed in vivo detailed structural features of the tumor boundaries. MSN further functionalized with a peptide (CF3) bound specifically bladder cancer cells.	[22]
Bimodal dual-modality peptide (CSNRDARRC)-conjugated NPs with iron oxide nanocubes and glycol chitosan derivatives (pMCNPs) DLS: 481.8 ± 8.7 nm	Bimodal imaging: FLI (OI by IVIS^®^ II Lumina using cyanine 5.5; exc 675 nm, ems 695 nm) and MRI (3.0 T)	In vivo: subcutaneous injection in nude mice of tumor canine K9TCC cancer cells incubated with NPs. Incubation/visualization time: 24 h. Ex vivo: analysis of major organs.	Development of novel MRI and NIRF dual-modality NPs. pMCNPs showed preferential accumulation and longer retention in small tumors (useful as 3T MRI contrast agents). pMCNPs acted as therapeutic agents using vinblastine.	[66]
Bimodal human serum albumin-MNO_2_-chlorin e6-NPs (HSA-MnO_2_-Ce6 NPs) DLS: 118.6 ± 8.1 nm Other NPs for single imaging technique: HSA-MnO_2_ (18.5 ± 4.8 nm) HSA-Ce6 (112.8 ± 7.4 nm)	Bimodal imaging: FLI (OI by IVIS^®^ Lumina; exc 675 nm, ems 710–900 nm) and MRI (7.0 T)	In vitro: mouse MB49 cancer cells In vivo: orthotopic bladder cancer model obtained by MB49 cells injection; NPs were administered i.v. Concentration: from 0.05 to 0.4 mM. Incubation/visualization time: 12 h. Ex vivo: analysis of excised major organs.	Excellent bladder tumor-targeting property of HSA-MnO_2_-Ce6 NPs. PDT with HSA-MnO_2_-Ce6 NPs showed therapeutic efficacy and significantly prolonged the lifetime of mice.	[67]

**Table 4:** Studies focusing on imaging bladder cancer tissue or cells using NPs or NP-based compounds for imaging techniques different from FLI or MRI. Studies are listed chronologically from the earliest to the most recent ones. exc, excitation; TEM, transmission electron microscopy.

Nanoparticles	Imaging Technique	Experimental Design	Main Results	Ref.
Two-sized oxygen nanobubbles (ONBs) TEM: 400 and 800 nm	HyperSpectral Dark Field Microscope (HSDFM)	In vitro: mouse MB49 cancer cells. Ex vivo: subcutaneous injection of MB49 cells in mice and treatment with ONBs. Excision of tumor and analysis of wax-embedded tissue slices. Concentration: 100 µg/mL. Incubation/visualization time: 4 days.	ONBs are suitable for in vitro imaging in single cells and ex vivo imaging in bladder cancer tissues thanks to the intense scattering signal.	[68]
Gold nanostars coated with silver and silica (AuNS@Ag@SiO_2_)	Surface-enhanced Raman scattering (SERS) in vivo, but visible ex vivo with multiphoton microscopy (exc 800 nm)	In vivo: mice were injected subcutaneously with MB49 cancer cells; after tumor development, NPs were injected i.v. Concentration: 3.3 mg/mL. Incubation/visualization time: 24 h. Ex vivo: analysis of excised tumors	Nanostars accumulated in the tumor but not in the healthy tissue.	[69]
Hyaluronic acid modified and liposome-coated IR1048 NPs (HAPO-1048 NPs) TEM: 135.2 nm	Optical coherence tomography angiography (OCTA) and photoacoustic (PA) imaging NIR-II region two peaks around 930 nm and 1100 nm	In vitro: Luc+ UMUC3 cells. In vivo: HAPO-1048 NPs were injected i.v. in an orthotopic murine model of bladder cancer. Concentration: 200 µg/mL. Incubation/visualization time: from 3–72 h Ex vivo: analysis of excised major organs.	HAPO-1048 proved to be a NIR-II photothermal agent for CD44-overexpressing bladder cancer, showing strong NIR II optical absorption, preferential tumor targeting, excellent biocompatibility, and high PTT efficacy.	[70]

## Data Availability

Not applicable.
